# Syphilis: Its Materials and Forces

**Published:** 1875-04

**Authors:** Z. Collins McElroy

**Affiliations:** Zanesville, O.


ARTICLE II.

### SYPHILIS: ITS MATERIALS AND FORCES;

### WHAT THEY ARE, WHAT THEY DO, AND HOW THEY DO IT, IN THE PRODUCTION OF THE PHENOMENA CALLED SYPHILITIC IN LIVING HUMAN BODIES; AND OUTLINES OF ITS CLINICAL MANAGEMENT.

BY Z. COLLINS McELROY, M.D, ZANESVILLE, O.

(From the Proceedings of the Zanesville Academy of Medicine, Zanesville, O., March 11, 1875.)

My first acquaintance with syphilis was in the "Venereal Wards" of the "Pennsylvania" and "Blockley" Hospitals in Philadelphia, and dates back nearly thirty years. I had heard before commencing the study of medicine, of the "bad disorder," with its "blue bores" in the groins, sore throat, and skin eruptions, and I had read in the newspapers the "important announcement" of "Drs. Humberg & Son," who ran "Lock Hospitals" in several large cities for the "cure" of the "afflicted" in "that" and other "ways."

But in the "Blockley," as a medical student, I was brought face to face with it; examined the sores, fungous growths, eruptions on the skin, etc., etc., of the patients there; heard the "clinical lectures" on the subject; watched the treatment; read "Hunter on the Venereal," "Ricord," and "Vidal," and, later, "Bumstead"; studied "Cullerier's" atlas; and carefully read the contributions to the medical press during my time, down to "Hutchinson" and "Drysdale"; and during my professional career have had some cases to manage, and have had many opportunities to study it, which were not neglected. I was most thoroughly taught, and as thoroughly believed, that "syphilis" was a veritable "disease," and no mistake.

There came a time, however, in my professional life, when the whole subject of "diseases" was overhauled, resulting in some important modifications of my creed in regard to sickness, which, of course, included syphilis.

In regard to it, I faced the alternative of becoming a skeptic, and rejecting much I had been taught, and up to this time believed, regarding its mode of operation, pathology and remedial management; or accepting what I cannot help regarding as a plain, common sense understanding of all the facts in regard to it, viz., that while the train of phenomena in living human bodies called syphilis, can, in our time, nearly always be traced to contact, it had a beginning which was not so, which might be repeated. That it was not, *sui generis* (of its own kind) a condition always the same ; and that remedial proceedings had some better foundation than empiricism. And finally, that it had near relations to a large group of so-called "diseases," whose sequelæ were characterized by permanent modifications, and losses of the natural structural arrangement of the material of living bodies, upon which natural or normal function depends, with, consequently, modifications and losses of function as these changes of structure progress.

What these teachings were, it is the purpose of this paper to state.

That to which the phenomena of so-called syphilis is due is designated "a specific virus or poison;" always communicated by actual contact or inoculation, (contagious or infectious.) The "virus" is supposed to be composed of, or contain, "germs," which, when introduced into an uninfected body, hypodermically, develop the "disease." It is certainly a matter to be noticed that it must be introduced otherwise than through the stomach to develop the disease. And whether it is directly through the external integument, or internal mucous membrane, it is, to all intents and purposes, "hypodermically," *i. e.*, through the investing integument.

I cannot help regarding this explanation of the modus operandi of any cause of any so-called "disease" as a most mischievous and misleading one. A "germ," according to Dunglison, means "the rudiment of a new being, not yet developed." "Germ force" means

"plastic force;" and "plastic force" means "the generative or formative power in organized bodies;" or, simply, "that which serves to form." Webster defines "germ," as, "In botany, the ovary or seed bud of a plant." "Plastic," as having the power to give form, or fashion, to a mass of matter. Does the so-called "syphilitic virus" do anything of this kind? It seems to me it does not. What it does is exactly the reverse, viz., modify or change, more or less completely, or entirely break up, existing "forms," or molecular arrangement of material in a living body, or structure. And its power, or force, everywhere else in nature than in a living body* is just *nil*, nothing. And, as function is altogether dependent on "molecular forms of structure," or the special molecular arrangement of material in living structure, as they are modified, changed, or lost, so is function modified, changed, or lost, whether by the "force stored up" in the "syphilitic virus," or any other of the numerous modes of force, "stored up in material" which changes, modifies, or breaks up the molecular arrangement of material in any living body. No existing function of a living body is bettered, or a new function added, in consequence of the work of the so-called "syphilitic virus." The sole effects of any of the modes of force "stored up" in material, which, in "living bodies," changes, or modifies, or breaks up the special structural arrangement of material in them, is "destructive,"—not "formative," or "constructive." Their materials are not "plastic"; nor is the force stored up in them "formative." And the application to any of them of the term "germ" is a misnomer, and mislead-

* Hunter's conclusions that syphilis could not be communicated to any inferior animals, proves to be incorrect. Turenne, in 1844 (France) succeeded in infecting monkeys. That rabbits, cats and horses could be infected from the monkey. His experiments lead to the idea of "syphilization," from the fact that inoculations became weaker and weaker as they were repeated; and a point was reached at which no further sores could be produced. His conclusion was, that prolonged inoculation ultimately resulted in "protection." In what way will be shown by and by.

ing, without new or modified definitions of the word; for the work of the builders of a house cannot be the same as the work of those who take or tear it down.

Rindfleisch, in the brief introduction to his work on "Pathological Histology," declares that there is not, in the whole range of pathology, a single lesion, or modification of structure, no matter to what cause it is due, which is not exactly similar to the changes of structure wrought by age or time.

The "germ," or "egg," or "cell" part of the so-called "virus" or "poison" of so-called "syphilis," is, therefore, altogether an assumption; for nobody has ever demonstrated a "cell," or a "germ" in the material, either with or without the aid of the most powful microscopes, or with any other treatment, physical or chemical.*

As it can be procured from syphilitic sores the virus is always blended with other products of the decay of tissue, and has, perhaps, never been completely isolated. In as clear a state as I have ever been able to obtain it, it presents to my eye, under a fair microscope (Ross, London) a homogeneous aspect all the time; not a "cell," or "germ," or "granule" was ever found, except such as are common to all sores, or suppurations. It appears, in fact, to be a homogeneous albuminous fluid, not at all unlike the "lymph" in small-pox, or vaccine pustules, plus common products of decay.

These facts and considerations forced me to abandon the "germ" concept concerning it.

But still the facts in regard to the fluid, or so-called "virus" of syphilis remained, and must be explained, or I must drop back into the old grooves again. The syphilitic fluid was a fact, as much so as that of small-pox, or vaccinæ; and inoculation was a fact; and the sequelæ

* A very recent European authority claims to have demonstrated the presence of "granules" in the virus of syphilis, and that its power for mischief in the living body resides in them.

were facts, that have occurred for centuries, and will probably recur for centuries to come.

What is the material of the "syphilitic virus"? what are its sources? and what is its chemical character?

The material is identical with that of living flesh, or possibly of the material of food arrested in its path to living flesh,* though the fact that the virus contains no pus globules must be received as evidence that its source is living tissue, or the materials of tissue, on its downward and outward career of organization. It is certainly from one, or the other, or partly from both; and it seems probable that it is at times from either or both; and if so, would account satisfactorily for the difference of results, which has led to the hypothesis of the "duality"—the double nature of the virus, as determined by the differences of results in different living bodies, *i. e.*, the soft and hard chancre, so called.

It is a matter for remark that in a large number of works on the subject I have consulted, no description of the physical appearances of the so-called syphilitic virus can be found, either as seen by the naked eye, or under the microscope. Descriptions of the appearance of the "sores" and "eruptions," "mucous patches," "buboes," etc.,—all modifications of the natural physical

* Prof. Saulsbury, of Ohio, verified by the subsequent researches of Dr. Lostorfer, of Vienna, has demonstrated the existence in the blood of some persons, under the influence of the syphilitic virus, of what Saulsbury calls "syphilis corpuscles," but not named by Lostorfer, who only claimed that he could, and did, by their presence or absence, determine syphilitic from the blood of uninfected persons, without knowing anything of its source. The discovery of Saulsbury was, and is, wholly neglected; while that of Lostorfer was denied by some of his cotemporaries, and in subsequent tests failed. It is probably certain both parties described what they actually saw under high microscopic powers. Had there been, or were there now, any "specificity" in syphilis, then appearances would be uniform in the blood of all infected persons. Efforts were made to fix the shapes and dimensions of "cancer cells," with like failure. As there can be no "specificity" in he ever changing forms of material in organic life, all efforts to fix them with exactness must of necessity fail, as they have failed in regard to syphilis and cancer.

appearances of the tissues—are abundant, with plenty of illustrations, plain and colored.

Simon (Chemistry of Man) states, that the syphilitic virus contains no pus globules; and beyond this brief allusion to its physical character—and nothing in regard to its chemical constitution—I have been unable to find anything in systematic works, or in organic chemistry, on the subject.

Exact statements in these respects are certainly very desirable, though not of sufficient importance to arrest further investigation until they are supplied, for the virus is certainly an albuminous fluid, or lymph, containing nitrogen, sulphur and phosphorus, difficult to isolate, certainly so in my observations, for chemical treatment, or observation and experiment.

Its exact chemical structure is, therefore, for the present, more or less shrouded in mystery; though its solution is merely a question of time. And by its chemical nature is meant, not alone its ultimate elements, for these are probably well known, but something of the mode in which they are combined in the virus. Hitherto attention has mostly been directed to the study of its morphological changes, but its chemistry must sooner or later engage attention, when its mystery will be cleared up.

The important fact in regard to it is that in its chemical complexity it stores up a mode of force, statical, possible or passive; which in living human bodies, becomes force dynamical, active or actual; expended chiefly in modifying, changing, or destroying the special molecular arrangement of material, or "the forms of organic structure," in living bodies on which natural function depends. And as they are modified, changed or lost, so is function changed, modified or lost. And the sole difference between the virus of syphilis or of gonorrhœa, as well as that of small-pox, and the whole catalogue of so-called "eruptive fevers," as well as that of "cutaneous diseases," is their speed and the extent to which they modify, change or obliterate the natural molecular arrangement of material in living tissue. And just now clinical observers and pathologists are working out the extent of the changes effected by it in nerve tissue and viscera.

The sharply defined line heretofore separating physiology from pathology is being slowly but surely obliterated. The very titles of some recent works are indicative of this change, viz., Pathological Histology, *i. e.*, the formation and development of abnormal organic textures. And the changes of the structural arrangement of material in living bodies, now designated and studied as "organic diseases," are declared by Rindfleisch to be "precisely similar to those which occur as a consequence of age."* That which is now studied as a separate block of knowledge under the name of pathology, once brought fairly within the acknowledged domain of physiology, it may be only on its outer borders, the restraints and hindrances to the study of morphology on its naked facts will disappear, and the new order of things will certainly clear up many now obscure points in so-called pathology, and syphilis certainly be one of them.

Such, it seems to me, after careful examination, are the naked facts, as now understood and taught by the profession in regard to the material of the virus of syphilis, so called; its sources, and its chemical and physical characters. Not much of a showing, to be sure, but nevertheless, just what there is to show, on the changes which it effects in living tissue—human tissue—and it must by remembered that it makes none on dead tissue, nor anywhere else in organic life, except in monkeys, and through them a few inferior animals, and "therapeutics," the literature of the profession, is full and extensive.

What is the virus of syphilis? and, placed in contact with living tissue, what does it do, and how does it do it? It is composed of the material of the body or bodies of those from whom it is derived. There is no other source

* Rindfleisch, Path. Hist., New Syd. Soc., p. 2.

from whence it can come or be derived. In its peculiar chemical condition, its stores up force, which, when introduced into other living human bodies, hypodermically, changes or modifies the molecular arrangement of their materials, as well as physical contour, followed by modifications of function, and remotely by loss of natural structural arrangement of material or "forms of organic structure," and consequently, loss of function and death.

That is, in my estimation, a technical though provisional definition of the so-called syphilitic virus, which includes everything pertaining to its material, chemical nature, and results, when introduced into a living body for the first time, and allowed to do its work without check or restraint from remedial agents, management, or mode of life.

In the study of what it is, what it does, and how it does it, if attention is confined to it alone, without reference to similar phenomena elsewhere in organic nature, correct solutions of its problems are not likely to be reached; for the mode of operation of the syphilitic virus in living bodies is not *sui generis*, nor alone, but takes place in obedience to well defined laws; and is precisely analogous to many other processes in both the vegetable and animal realms of nature. Pollen, in the vegetable world, is an analogous substance, storing up force in its material, which, applied under certain conditions, modifies organic structures in growing vegetables. "Crossing" breeds, illustrates, in animal life, the power ordinary inorganic material in special organized conditions has of storing up force to modify subsequent organic structures. Reproduction, multiplication, and perpetuation of organic life, animal and vegetable, is all based on, and actually accomplished, by force "stored up" in material, to form, modify or change existing, or growing, or forming organic structures.*

* Force stored up. Perhaps it may aid in comprehending the conditions of force to state, that nothing is known of force except in two states, viz., force statical, or force potential, or force possible, or force passive,

It may not be unprofitable to study the work of a virus, so called, in modifying the structural arrangement of materials in living bodies, having simpler conditions than that of the syphilitic virus. Most of us in civilized life now-a-days, have both "to do and suffer" the simple operation and results of vaccination. A little clean albustored up in material; to become, with chemical changes of structure, or mechanical changes of position or structure, force dynamical, or force actual, or force active.

Thus: a piece of coal or wood, such as is ordinarily used as fuel, is material storing up force potential, or force possible, or force passive; to become, by combustion, which is simply chemical changes in its materials, *i. e.*, chemical decomposition, which force possible, potential or passive becomes, in the act of chemical decomposition, or combustion in the materials in which it is stored up, force dynamical, or force actual, or force active *i. e.*, heat, or simply motion by matter. And this applies to all force and to all matter, inorganic or organic. Chemically complex organic materials store up force potential, possible, or passive; to become, under certain conditions, force dynamical, actual, or active. I think this applies to organic structures themselves as fully as it applies to a piece of coal or wood, and that the evidence to this effect is as complete as it is possible for demonstration to make it. Each special structure, histologically, stores up force potential, possible or passive, to become, by decay, physiological or pathological, force dynamical, actual or active; and the force stored up in living organic tissues, when it is changed from force potential, possible or passive, into force dynamical, actual or active, is what is called "function" in organic life.

Chemical changes in brain matter, modified or changed in the structural arrangement of material, is announced or made known by changes of function. Mental equilibrium gives place to delirium, or the reverse, coma; or, again, to what is called insanity, dementia, or paralysis, etc., etc.

And it applies to medicines. Medicines, drugs and medicines, are materials, storing up, in virtue of special molecular arrangement of matter, force potential, possible, or passive, or statical; to become, when introduced into living bodies, force dynamical, actual, or active; and is manifested by advancing, modifying, or retarding the rate or pace of molecular changes in the materials of living tissue; and not in the twenty to forty different modes designated in existing classifications of medicinal agents.

Thus: alcohol does not "stimulate," *i. e.*, advance the pace of molecular work, or changes in the materials of living flesh. On the contrary, the decline of temperature in most cases following its introduction into a living body, demonstrates that the speed of molecular work is retarded, and the changed or modified functions equally demonstrate that structure, and motion in structure, have been modified.

minous lymph on the point of a lancet, knife or needle ; a few scratches of the skin, just sufficient to penetrate the cuticle, and the operation is complete. In forty-eight hours a reddish spot appears at the place of introduction, if the material, operation, and individual operated on, has been right—unprotected. At the expiration of a hundred hours a decided change has occurred, not alone at the spot of introduction, but the whole body participates in it. There is some elevation of temperature of the whole body; and at the point of introduction an opaque spot, whitish, surrounded by a zone of redness, more or less marked. At the end of two hundred hours, a distinct pustule has arrived at maturity, filled with a clear lymph, which speedily becomes floculent, desiccates, forming a dark brownish crust, which may be detached, or falls off, between the twelfth and eighteenth days after the introduction of the virus or matter. At the spot on which the crust or scab had formed, there is left a more or less decided excavation in the skin, upon which crusts successively form, as they are detached, until the final healing takes place ; and at the end of from four to six weeks, or in some cases longer, the color of the skin has become natural. But there has been left a scar or mark, more or less circular, or it may be altogether irregular, with a number of small pits or excavations in it. And the object generally of this proceeding is to gain immunity from the effects of another virus called small-pox.

This picture represents only "straight" or normal cases. And it must be borne in mind that such are comparatively rare or few in number ; for there are endless variations in the amount of constitutional disturbance, progress of pustule, and scars resulting, after the operation of vaccination. The operation may have other purposes than protection against small-pox, viz., to hurry up the progress of so-called whooping cough, indolent enlargements, etc. Nor is it altogether free from serious consequences. Within my own scope of observation, death has ensued a good many times as a sequel to vaccination. These results were explained by the assumption that "impure" matter had been used, or the person was of a "scrofulous habit," etc. These are now regarded as conclusions drawn from erroneous premises, as I hope to be able to show before I get through with the subject.

After vaccination has been successfully accomplished, in a large majority of cases, but not all, small-pox lymph, and vaccine lymph may be introduced into the body with impunity, or at least without noticeable results, for a length of time somewhat variable, sometimes through life.

What has taken place in the body of a human person successfully vaccinated? What is vaccine lymph? In seeking a solution to the question, What is vaccine lymph? it is not needful to assume anything as a starting point, nor anything in regard to its containing "germs" or "organisms." Better to study it and its consequences in the living body on their facts. Vaccine lymph, like syphilitic lymph, small-pox lymph, etc., is identical in material with the material of the body from which it has been derived, and differs from all other fluids of the body only in the molecular arrangement of materials. Chemical complexity, everywhere and under all circumstances, stores up force, statical or passive; surrounding materials and conditions determining the character and extent of the modifications it will effect in them when it becomes force actual or active. Thus, the stored up force in the lymphs or viruses, which, in living bodies, gives rise to the several phenomena of small-pox, vaccinæ and syphilis, would appear elsewhere in nature as heat only. The changes effected on the exterior of living bodies by the small-pox and syphilitic viruses are obvious enough. What has occurred in the interior is not quite so plain, though it is absolutely certain that it is in the molecular arrangement of materials. From the activity of the processes in some of them, as small-pox, as evidenced by increased heat of body, the molecular changes may be brought about in some such a way as a small flame applied to a sheet of cotton batting communicates its own mode of motion to the whole mass. Flame is force actual, active, which has been stored up in the materials undergoing combustion. Cotton stores up force potential, or passive, or statical, which becomes actual, or dynamical, in the act of combustion. Flame applied to cotton propagates itself by actual contact. Therefore, flame, cotton and contact are conditions for changing the molecular arrangement of materials in cotton. Cotton cannot be burned, or charred, twice, until the materials disintegrated are recombined again. So, of the results of vaccination, small-pox, etc., in living tissue. What has been done once cannot be repeated until the tissues have been remodeled again, so to speak. And but too often in living bodies the molecular arrangement of material has been, like the burned cotton, wholly lost, function abolished followed by death. For there is no greater certainty connected with the problem of life than that function is dependent on the special molecular arrangement of material in each organic tissue or viscus ; and as this is modified, changed or lost, so is function modified, changed or lost ; and for certain structures that is death to the whole organism.

Vaccine lymph, as an ordinary result, does not effect such changes in the molecular arrangement of the materials of living structure as to perceptibly change function ; for if it did it would not hold its place a moment among remedial or prophylactic measures.

That such is the modus operandi of the virus or lymph producing the several phenomena called vaccination, small-pox, gonorrhœa and syphilis, is shown in many ways, as the acceleration of the general movement of material—fever—during their progress, as evidenced by the clinical thermometer, by the changes in the appearance of the cuticle and cutis beneath, at points of entrance or pustules, where the entrance has been hypodermically effected, and at other points, and eruptions, etc., elsewhere.

As all organic life, animal and vegetable, is made of one stuff, information may be very properly sought among the simpler forms of vegetable life, to throw light upon the complexities of animal life. Thus, something may be learned in regard to "storing up" force for special and definite purposes from the vegetable world. A vegetable seed is—

1st. The necessary material;

2nd. To store up the requisite force;

3rd. To form the structures of the new plant up to the point of independent existence; or the appropriation of inorganic material to its further growth and evolution.

Coming back again into the domain of animal life, the egg furnishes another striking illustration of "storing up force" in the requisite material for exactly like purposes as a vegetable seed, except that the young animal, after entering its stage of independent existence, requires organized material, in certain chemical conditions, for its further growth and evolution, but always "germinal matter," *i. e.*, material in which some previous animal or vegetable existence had "stored up" the material and force for its own preservation, perpetuation or multiplication.

The real difference between small-pox, vaccination, syphilis and gonorrhœa—and in the same class are included scarlet fever, measles, whooping cough, and the whole catalogue of "skin diseases"—is not "specificity," but mode and speed in changing the molecular arrangement of the materials of living tissue. And the mortality attending the processes of each follows very closely the speed, as determined by the temperature.

Small-pox, scarlet fever and measles, which are accompanied by the highest ranges of temperature, and, therefore, the highest speed, have the largest death rate. Thus, vaccination has a not very variable speed, its work in a living body being accomplished in something near a month of time, seldom less, often more. Small-pox has a higher speed and longer duration, extending over nearly or quite three months. Measles, apparently shorter, yet the disturbance of temperature extends over one to three months. Scarlet fever has, perhaps, the highest speed, and extends over two or more months. Whooping cough slower than either, extending, at times, over a whole year, as determined by changed functions. Syphilis and gonorrhea have a still slower speed, with indefinite duration, syphilis, in many instances, extending over the remainder of life.

At certain stages, the temperature of syphilis, as well as of gonorrhœa, is from three to five degrees above natural; but in the main is nearly or wholly natural, though the change in the molecular arrangement of structure still proceeds, as definitely demonstrated by changing functions, and physical appearances of structure, within the scope of vision.

The whole catalogue of "chronic," *i. e.*, slow moving, "skin diseases," eczema, prurigo, etc., are characterized by little or no disturbance of temperature, and are, therefore, of indefinite duration, and have many very close points of resemblance to syphilis. It is, therefore, apparent from even a casual study of the phenomena of these several so-called "diseases," that they are due to modes of force, and the velocity at which they act, rather than to "specificity," or any essential difference in materials, or the force concerned in their movements.

The so-called "syphilitic virus," it seems to me, has been shown to be material identical with that of the living body from which it is taken, and differs in no respect from other fluids, or any special fluid, of an uninfected body, than in the molecular arrangement or structure of its material. That in this special molecular arrangement of material, there is stored up force statical, potential, or passive, which, when introduced into an uninfected living body, may become force dynamical, actual or active, communicating its own mode of motion, or, perhaps, more properly speaking, modifies the movement of material on its path to living flesh, with the result of forming, so to speak, syphilitic tissue, which differs very widely from normal tissue in living human bodies.

I find little difficulty in getting a conception of how it is done, by studying other phenomena, chemical and dynamical, in the inorganic as well as organic realms of nature. Everywhere, bringing together two separate materials, storing up in their chemical complexity force statical, possible or passive, under certain conditions to become force dynamical, actual or active, if there exist any chemical affinities between them, the result is a *tertium quid*, a third something, having no resemblance, physically, to either of its component parts. Thus, in colors, blue and yellow combined in certain proportions, form green. Sulphuric acid and oxide of iron, one a fluid, the other a solid, united, form a third something which presents none of the physical appearances of either of its constituents. Among organic materials such results are still more striking, as in photography. Coal tar, crude opium, Peruvian bark and alcohol by chemical manipulation yield the most varied results. But my favorite conception, based on the unstable chemical states of the materials actually composing living bodies, and the so-called syphilitic virus, is a minute flame applied to a mass of fleecy cotton, with the single modification in the matter of speed ; the cotton burning rapidly, in that respect resembling scarlet fever ; while the work of syphilis goes on at a snail's pace ; and when unmolested by treatment, or other external conditions, ceasing only when it has "no more worlds to conquer," no more tissue to disarrange.

The results, too, of remedial proceedings are very properly regarded as shedding some light on the problem. Medicines are materials, organic or inorganic, storing up force in themselves—*i. e.*, in their chemical complexity, or, molecular arrangement of material—which, in living bodies, have no other influence than a partial control over the physical movements of materials, as they ascend to or descend from their condition in living flesh. And this within a very narrow range, for if the physical movement of material is advanced beyond a definite limit, as with strychnia, or retarded below an equally definite limit, as with prussic acid, they are ranked as "poisons." Others, again, operating beyond these limits in advancing motion of materials, are regarded as "caustics"; or below, as "anæsthetics," or "poisons." These, and other like operating medicines, are ordinarily composed of materials natural to the body, in other and widely different states of chemical union. But materials altogether foreign to the tissues of living bodies, as iodine, arsenic, mercury, etc., in various combinations, like complex organizations of materials natural to them, may appear to advance, retard or modify the physical motions of materials composing them; but, in reality, universally, and necessarily, acting always in the interest of waste of existing tissue, and increase of the speed of eliminating effete products and material. Whether the ultimate results are in the interest of repair or waste, depends somewhat on quantity introduced in a given time, and condition of motion in materials of structures, thus, sometimes doing what is deemed desirable work, at others effecting changes not only not desirable, but actually damaging.

The leading therapeutic agents for the remedial management of syphilis are mercury, potassium iodide, caustics, salines, natural saline mineral waters, to which may be added, though not so recognized, hard labor in the open air. Or sometimes agents operating exactly the reverse, as iron, quinia, bismuth, mineral acids, and many other so-called tonics, etc., etc.

Now what do these several agencies do? Mercury, confessedly first in importance on the list, is an agent of tissue waste *per se;* is material altogether foreign to the tissues of the human body, and in the laboratory of life, so to speak, its force statical, or passive, is displayed as force actual or active, first, last, and all the time, in the interest of increased waste of living tissue, without much disturbance of temperature, and hence, is classed as an "alterative." There is, of course, a complex play of chemical affinities, as the result of the new conditions it introduces, difficult, and not now possible, to explain in detail; but the increased waste of existing tissue is the fundamental and important fact and result. And that is the precise reason why actual working practitioners, after experimenting unsuccessfully with other remedial agencies, invariably return to this imperfectly understood, but, in the main, certain working agency, in their hands. For persons in otherwise good health, not engaged in hard wasting labor, it is the *sine qua non* of remedial management, if success is aimed at.

Potassium iodide, the second on the list in importance, is, like mercury in one respect, half and half a foreigner to the tissues of the body, yet in good company, for the potash is a natural and essential constituent of living tissue; one of whose uses is to become a sort of vehicle, or car, to convey the results of interstitial decay of the tissues to their appointed places of egress from the body. Iodine, however, like mercury, is a wasting agent, *per se*, though operating slower. For persons in whom tissue making and repair is below par, it very properly takes the place of mercury in any of its various combinations.

Then come the salines—wasting agents, but essential constituents of living tissue in definite proportions. Introduced in excess, they become wasting agents, with less power than mercury or iodine, with the exception of tartar emetic. Sea voyages, natural mineral waters, travel, etc., etc., are auxilliaries, indispensable under some conditions, in remedial management of syphilis, viz., when the processes of waste and repair are performed too sluggishly. Caustics, too—agents whose force, statical, passive, or stored up, when applied to living structure, is manifested as force, actual or active, too rapid to preserve forms of organic structure, which are accordingly lost—have their place and uses in the remedial management of so-called syphilis; and are essential adjuncts of effective treatment to break up, and secure the complete disintegration of tissue altogether out of natural structural forms; a necessary procedure to save the lymphatic system, whose function will be explained presently.

Neglected or not understood remedial agencies, are hard labor in the open air, exposed to the sun and weather.*

There is no more efficient ally of the syphilitic force dynamical, or actual, in the living body, than sedentary habits of life; for, in such, there is no proper rate of waste and elimination kept up; so that the work of changing forms of organic structure goes on without much opposition.

Then, again, there are conditions of living bodies in whom the work of repair is reduced to its minimum; while the rate of waste is in excess of even that of normal repair, in which the so-called tonics, agents which, in their stored up, or statical force, supplement, as it were, the deficient natural forces of repair, are not only demanded, but are essential to recovery, or continued life.†

* Dr. Alfred Ball, Surgeon of U. S. Volunteers, during the late war, informed me that when the soldiers lay in camp any length of time, he would soon have a large number of cases of syphilis on hand. Orders to march, especially if the duty and exposure were severe, was invariably followed, not by relief, but apparently good recoveries. In his own language, "We never heard much of syphilis during long and severe duty, but whenever we went into camp they were sure to turn up. But the cases that appeared in camp were not relapses from former conditions, but were new cases." Hard duty seemed to relieve them as if by magic.

† Syphilization, orginally suggested by Turenne's experiments on inferior animals with the virus of syphilis, was, perhaps, first carried into actual clinical practice by Sperino, of Turin. Prof. Boeck, of Sweden, while traveling in Italy in 1851, had his attention drawn to the results of Sperino's practice; and has, since that time, devoted himself assiduously to the subject at his home in Christina, and is now the leading authority in regard to it. Though in his hands, as it had been in Sperino's, it was a strictly empirical clinical measure, it is an effort in the right direction, and has a

It is thus seen that the employment of remedial agencies comes under the dominion of definite laws. Mistakes may be, and perhaps are, habitually made by the most expert prescribers, but the fault is not in the solid, scientific basis. Its aim is to "saturate," so to speak, the system, and to attain by repeated inoculations, the end actually accomplished by the virus of vaccinæ, as well as those of small-pox, scarlet fever, measles, etc., *i. e.*, "protection," or "immunity," from future effects of these viruses after one "attack."

What syphilization really accomplishes, it seems to me, is to get up, and keep up, the requisite speed, and mode of "changing molecular forms of structure of living bodies," at which they are not essentially damaged during the process, *i. e.*, admitting the future performance of function, not perceptibly unnatural, as after small-pox, vaccination, etc., etc.

It seems to me that the results attending or rather succeeding the introduction of vaccine virus in unprotected persons, or persons into whose bodies it has never been previously introduced, who are suffering from whooping cough, at certain stages of the complaint, points out the ultimatum of the clinical management of syphilis. That vaccine virus, which has a definite, and not very variable speed, in doing its work in the living body, communicates its own speed to whooping cough, which has no definite speed, like syphilis, so that when the work of the vaccine virus is complete, the whooping cough has ceased, I have verified, again and again, during my professional career. In fact, it is one of our most certain clinical measures in whooping cough, at particular stages. But we are not without some positive clinical evidence on the point I wish to make, viz., that syphilis can be hurried through its stages, so as to save the structures, and gain immunity from it in the same way as small-pox, measles, etc., are self-protective. Thus: Prof. P. J. Farnsworth, of Clinton, Iowa, reports (*Cin. Med. News*, Dec., 1874, pp. 556,) a case of a gentleman who had the "copper discoloration of skin, loss of hair, pains in the bones," etc., who, while under treatment for "secondary" syphilis with no encouraging success, had the peculiar eruption of small-pox suddenly make its appearance, and pass through its stages in a mild form. When he had recovered from the small-pox, all traces of syphilis had disappeared. Twelve years later he saw the patient, who had subsequently married, and had offspring, with no evidence of syphilitic taint in either himself, wife or children. Here, small-pox had communicated its own rate of speed to syphilis, so to speak, both ending at the same time, without material damage to structure, as evidenced by his subsequent life during twelve years.

It seems to me, therefore, that the ultimatum of the clinical management of syphilis, will be by some mode of "force," "stored up," which will hurry it through its stages in the briefest possible time, and thus make it self-limited, and self-protecting, as small-pox, measles, etc. ; and when seriously sought after, will, sooner or later, most likely, be found. The absence of, nor in the variation of law, but imperfections of the prescriber in interpreting the actual condition of patients, behind the objective symptomatology presented for his study. Still, when conditions are more conspicumost successful clinical management is better explained in this way than any other now known, as will be shown by and by, when its clinical management comes under notice.

Sir Wm. Jenner, in his just published address on taking the Chair of the Clinical Society of London, (*Med. Times and Gazette*, Feb. 20, 1875,) says: "When any acute specific disease is epidemic, the public—the educated public—call loudly for a cure; and too often, I think, members of the profession call out as loudly, 'Eureka!' Now it seems to me that there are no grounds for expecting that a cure will ever be found for diseases of this class—(small-pox, measles, scarlet fever, etc.)—*i. e.*, for expecting that a drug or medicinal agent will be discovered, capable of arresting the progress of the organic changes which, set in motion by a special cause, and following each other in definite and ascertained order, constitute what we call an acute specific disease. For, in place of being diseased actions, these several organic changes, the evidence of which we call symptoms, are, as far as our present knowledge extends, processes, the first of which being called into action by some external cause—*e. g.*, the poison of the disease—are essential for the restoration of the intimate organic changes to the order and intensity which constitute health.

"I may illustrate my meaning thus:—Each of these diseases may be likened to a single fit of ague. We administer drugs to prevent the recurrence of the fit, but we do not cure the fit itself; the cold stage having commenced, and attained a certain instensity, the hot and sweating stages are essential for the restoration of the balance of health. So with small-pox. By vaccination we prevent its occurrence; but when the first of the phenomena, which, following each other in certain and definite order, constitute an attack of small-pox, occurs, we know that there is no road to health, but by the sequence of changes the symptoms of which mark the several stages of the disease; and to my mind it is very questionable whether if it were possible, by the administration of a drug, to arrest the essential changes which constitute any of these stages, health would be the result. And what is true of small-pox in this particular, seems to me to be equally true of all the acute specific diseases."

Sir Wm. does not include syphilis among "acute specific diseases," perhaps, because its course is so slow—left to itself, or imperfectly treated. But it falls in the same category, and when any mode of "stored up force" becomes available to hurry it through its changes in a definite and limited time, then will therapeutics be the master, which it falls very short of being now.

I well remember the disgust excited in my mind by Prof. Boeck's practice of syphilization, when I first read about it, twenty years ago. Syphilis ously studied instead of "the disease," and "stages of the disease," there will be fewer blunders.

The purpose behind any and all remedial agencies can be clearly and definitely stated. In order, however, that they may be more clearly comprehended, it may be well to review the phenomena in the light in which they have been presented.

The train of phenomena called syphilis is brought about by the introduction into an uninfected living human body, hypodermically, of material, in such a state of molecular organization, as to store up force—statical, potential, possible, or passive—to become force dynamical, or force actual, or active, for its own multiplication and perpetuation amidst the complex materials of living human bodies, and thus effecting modifications of, or completely changing the structural arrangement of materials, on which natural function depends, in the various organic structures composing an individual living human body.

Unless purposely and knowingly inserted, the fact of the introduction of the syphilitic virus into a living human body by chance or accident, is, and can only be known, by changes, or modifications of structure, generally, though not always, at the point of its introducseemed to me to be the most loathsome of all "diseases," and the idea of multiplying and perpetuating it in that way in living bodies, seemed to me to be the very height of absurdity, if not actually criminal. I then gave Prof. Boeck and his syphilization a most hearty and unqualified condemnation. But since I have been "re-constructed," medically, he, and his practice, appear in a very different light. Syphilis is not more loathsome at any stage then small-pox, and if it had the speed and limited duration of small-pox would cease to be a puzzle to actual working practitioners, who have to deal with it, just as small-pox has. But the therapeutic problems in the two cases are exactly opposite to each other. In the remedial management of small-pox, practitioners are more concerned in slowing its speed than with any other feature, while in syphilis the problem is to hurry up speed. This, syphilization does to some extent, but its nine to twelve months duration is too slow for the present state of civilization. Vaccination out of the way, the small-pox virus might become the "spe. cific" of syphilitic treatment, as quinia is of ague. But vaccination, as now insisted on in early life, blots this chance out at later periods.

tion; first, the chancre, and certainly there can be but one conclusion as to whether a chancre can exist without changes in, or a total loss of, the structural arrangement of material in living tissue. For these must occur that a chancre, so called, can have an existence. Here comes in the caustic and mercurial treatment, whose object is to "stamp out," so to speak, the multiplying capacity of this special material; and if accomplished before the lymphatic system has become deranged, the relief, or restoration to normal condition again of the body, is complete at once. But, as often happens, the lymphatic system becomes involved, and then modifications of tissues having the highest rates of repair and waste, as the skin and mucous membranes, speedily occur, and they no longer present natural objective appearances. Sometimes the lymphatic system is overwhelmed at the initial point, and the result is, the glands nearest to the local point of introduction lose completely structural forms, repair is wholly arrested, waste goes on at a pace too fast to allow the debris to be removed by natural means and through natural channels, and suppurations, so called, are established, and the tissue of the gland is speedily broken up.

The order of the disarrangement of the tissues will be in the order of their stability, and the velocity of repair and waste in them. After the skin and mucous membrane, and lymphatic glands, the "mother of bone"—the periosteum—next shows departures from natural structural arrangement, followed in due time by permanent changes in the bones themselves; first noticed about points only covered by cutaneous tissue, though not likely confined to them; then, the smaller and softer bones about the nose; then, the interior structures of the globe of the eyes; and finally, the central mass and connections of the nervous matter—the brain and nerve masses and viscera—each suceeding modification, or loss of structural arrangement of matter, announced by changed or lost functions and physical appearances. Now, how are these modifications of the structural forms of the materials of living bodies brought about? Not by a "disease," but by adequate means to these ends; by force, passive, stored up in material, becoming force active, actual, or dynamical, in the midst of the chemical decompositions and recompositions certainly and ceaselessly taking place in human bodies, alive or dead.*

Living tissues, in detail, as well as in the total of each individual, are perpetuated from new material in obedience to orderly law, precisely analogous to the mode in which plants are perpetuated in the vegetable realm of nature, and not by a special police, or constabulatory force, as it were, in the so-called "vital force," the hypothetical means to which their preservation and perpetuation have been referred for so many centuries.

How are they multiplied and perpetuated? Precisely as wheat and corn, and all other vegetables, are multiplied and perpetuated—*i. e.*, by force stored up in material for that purpose, in the act of final decay in the performance of function, *i. e.*, a seed. But, in living animal bodies, the material in which the various structures store up the force for their own perpetuation, as they are disintegrated in the performance of their functions, it is the duty, or function of the lymphatic system to collect, and separate from the general debris of the structures, and unite it with the ingoing stream of new material, to make good the waste of structure in the performance of function. This is done just before the stream of ingoing material enters the right auricle on its way to the lungs. In the lungs this complex stream meets the inhaled gaseous atmosphere, when and there the physical, chemical or molecular changes as they may by preference be called,

* Thirty years since, my only sister died at the age of 21, quite suddenly, and therefore her body was not much changed from its nearly physical perfection during life, at her burial. The ground of the cemetery is now demanded for other uses. Opening her grave two weeks since, nothing was found, except portions of her osseous system, only dark lines in the soil, indicating the outlines of her burial case. The materials of her body got no rest in the grave, as they had had none during life.

take place which fits new material to become living flesh, capable of performing a function, chief among which is providing for their own reproduction and perpetuation from new material, as wasted in the act of functional decay. Whatever there is of "vital force" concerned in life, and living beings as complex as the human body, is to be looked for, and found, in the lymph; and, in the vegetable world, the "seed."

This understanding—explanation—of function, and play of matter and force, at once satisfies all scientific and moral requirements in accounting for all the actual phenomena of life, viz., personal identity, with changing material through life; and the physical death of the parent is a necessity that offspring may have individuality. It satisfactorily accounts for certain so-called "diseases," as scrofula, phthisis; tumors, from adipose to cancer; and for the physiological—histological and pathological—histological facts of normal life, and post mortem states of the tissues.

From these facts and considerations, and the inductions to which they necessarily lead, in regard to histogenesis and histolysis—play of matter and force in living bodies—it is not difficult to lay down general principles to guide the actual working practitioner in the remedial management of so-called syphilis, enough of detail being left to him to tax the best possible mental powers of each and every one.

They are as follows:

1st. For all in whom the processes of repair and waste of living structures is at its maximum, or standard of health, or near that, and in whose bodies there are certain structures, which have been so modified by the so-called virus of syphilis as to be incapable of performing natural functions, the indications are to waste them as rapidly as can be done without too much disturbance of the processes of repair.

To meet this indication, mercury in some form is the leading remedial agent, and most effective in vapor to the cutaneous surfaces.

2nd. With decreased and decreasing capacities for normal repair, mercury cannot be as safely employed as the potassium iodide, and salines.

3rd. When repair is manifestly below the normal standard, as evidenced by loss of appetite, weakness, and pallor of cutaneous surface, resort must be had to a mixed treatment, as potassium iodide in combination with so-called tonics; among which quinia will always hold a leading place, iron, bismuth, strychnia; and barks having peculiar properties—that is, storing up force particularly adapted to supplement the natural powers and capacity for repair, as columbo, gentian, black alder, prickly ash, poplar, cundurango, eucalyptus, etc., including cod oil.

4th. Where repair is much below the requisite pace, all wasting agencies must be laid aside, and all available means, hygienic and therapeutic, brought to bear to bring up repair as nearly as possible to the natural standard.

5th. In every condition, good food and every sanitary measure must be properly looked after. For living structures are not made out of nothing, but from the food eaten, and its character much influenced by surrounding conditions.

Previous and present health, occupation, situation in social life, etc., will have much to do, not only with diagnosis, but especially with prognosis, and consequently with remedial management. Active, hardy men and women, in young or middle life, will fare better, with and without treatment, than the sedentary, indolent, and poorly provided for, of whatever stations in life.

The purpose and aim of this memoir is to show that what is called and known as syphilis, can be understood in its entirety, viz., what is its cause, physical, chemical and dynamical; what it does in living human bodies, and how it is done; what remedial measures are proper in each individual case; what are improper; and that these things can be definitely stated, and definitely understood, without the scholastic furniture with which our books now clothe and surround it. And that the actual working practitioner who can emancipate himself from the shackles of the nomenclature and ideals now held in regard to it—syphilis—and will study it on its own facts, and prescribe its remedial management based on separate studies of each individual case, can rarely fall into error, or if so, it can only be temporary, as the results of treatment will soon point out any errors in the mind work given to it in the beginning.

In closing, I wish to acknowledge my indebtedness to the authors of the published works on the subject, past and present. I recognize in them careful observers, and cautious experimenters, with rare descriptive powers. Viewing the phenomena of syphilis so differently from all of them, there seemed to me no place in the body of my monograph, where the obligations I am under to them, could be acknowledged, save just here.

